# Elucidating the Role of OXPHOS Variants in Asthenozoospermia: Insights from Whole Genome Sequencing and an In Silico Analysis

**DOI:** 10.3390/ijms25074121

**Published:** 2024-04-08

**Authors:** Maria-Anna Kyrgiafini, Themistoklis Giannoulis, Alexia Chatziparasidou, Zissis Mamuris

**Affiliations:** 1 Laboratory of Genetics, Comparative and Evolutionary Biology, Department of Biochemistry and Biotechnology, University of Thessaly, Viopolis, Mezourlo, 41500 Larissa, Greece; 2 Laboratory of Biology, Genetics and Bioinformatics, Department of Animal Sciences, University of Thessaly, Gaiopolis, 41336 Larissa, Greece; 3 Embryolab IVF Unit, St. 173-175 Ethnikis Antistaseos, Kalamaria, 55134 Thessaloniki, Greece

**Keywords:** asthenozoospermia, oxidative phosphorylation, variant, sperm motility, biomarker

## Abstract

Infertility is a global health challenge that affects an estimated 72.4 million people worldwide. Between 30 and 50% of these cases involve male factors, showcasing the complex nature of male infertility, which can be attributed to both environmental and genetic determinants. Asthenozoospermia, a condition characterized by reduced sperm motility, stands out as a significant contributor to male infertility. This study explores the involvement of the mitochondrial oxidative phosphorylation (OXPHOS) system, crucial for ATP production and sperm motility, in asthenozoospermia. Through whole-genome sequencing and in silico analysis, our aim was to identify and characterize OXPHOS gene variants specific to individuals with asthenozoospermia. Our analysis identified 680,099 unique variants, with 309 located within OXPHOS genes. Nine of these variants were prioritized due to their significant implications, such as potential associations with diseases, effects on gene expression, protein function, etc. Interestingly, none of these variants had been previously associated with male infertility, opening up new avenues for research. Thus, through our comprehensive approach, we provide valuable insights into the genetic factors that influence sperm motility, laying the foundation for future research in the field of male infertility.

## 1. Introduction

Infertility is a significant global health concern, estimated to impact 72.4 million individuals worldwide [1]. Male factors are implicated in 30–50% of infertility cases among couples of reproductive age [2]. Male infertility is a multifaceted condition influenced by a combination of environmental and genetic factors [3], and it encompasses a wide range of subcategories, each involving various qualitative and quantitative sperm defects [4]. One prevalent cause of male infertility is asthenozoospermia, defined by the fifth edition of the World Health Organization (WHO) guidelines (https://apps.who.int/iris/handle/10665/44261, (accessed on 5 March 2024)) as having reduced sperm motility (<40%) or progressive motility below 32%. In this condition, although sperm are present in the semen, they lack the ability to move or progress sufficiently for the journey from the vagina to the fallopian tube. Consequently, fertilization becomes unattainable.

In the context of asthenozoospermia, the motility of sperm hinges on the availability of energy. Thus, recent investigations into sperm physiology have cast the spotlight on the mitochondrion, acknowledged as the cellular powerhouse. The mitochondrion plays a pivotal role in sperm function, impacting not only motility but also other critical aspects of fertilization, including capacitation, hyperactivation, and the acrosome reaction, thereby influencing overall male fertility [5]. Furthermore, current research highlights two primary metabolic pathways that contribute to ATP production for optimal sperm function: oxidative phosphorylation (OXPHOS) and glycolysis [6,7,8,9]. OXPHOS, the predominant metabolic pathway occurring within the mitochondria, is essential for proper sperm function, and disruptions in mitochondrial oxidative phosphorylation are posited to compromise normal sperm activity and especially sperm motility [7].

The oxidative phosphorylation system (OXPHOS) on the inner mitochondrial membrane consists of five enzymes organized into complexes I–V: NADH: ubiquinone reductase (Complex I), succinate dehydrogenase (Complex II), quinol-cytochrome c reductase (Complex III), cytochrome c oxidase (Complex IV), and H^+^-transporting two-sector ATPase or FoF1-ATPase (Complex V), which synthesizes ATP. In humans, these enzymes are mostly multimeric, with subunits encoded in both the mitochondrial genome (mtDNA) and the nuclear genome (nDNA), excluding Complex II [10].

However, despite the recent extensive research in the field, the molecular mechanisms underlying asthenozoospermia remain not fully understood in the majority of cases. Additionally, there is limited literature on specific mutations in OXPHOS genes that may cause asthenozoospermia.

Therefore, this study aimed to conduct whole-genome sequencing (WGS) in individuals with asthenozoospermia and those with normal sperm motility (normozoospermic), with the primary goal of identifying and characterizing variants within OXPHOS genes (both nuclear- and mtDNA-encoded) exclusive to asthenozoospermic men. By doing so, we sought to elucidate genetic factors contributing to the pathogenic phenotype associated with reduced sperm motility. It should be noted that the overarching objective of our research was to provide a comprehensive reference for future investigations into asthenozoospermia, offering insights into variants that potentially influence the functionality of the OXPHOS machinery and impact sperm motility. This endeavor involved an integrated approach that combines whole-genome sequencing with bioinformatics and in silico tools.

## 2. Results

### 2.1. WGS Results—Variant Calling and Annotation

After whole-genome sequencing, data analysis was conducted. Specifically, a comparison was performed between normozoospermic and asthenozoospermic individuals to identify unique variants present exclusively in one of the two groups. A total of 680,099 variants were observed exclusively in asthenozoospermic individuals, while 2,329,803 variants were found only in normozoospermic men. These variants were then mapped to 30,362 and 26,019 genes in normozoospermic and asthenozoospermic males, respectively.

For the purpose of this study, only the variants identified in asthenozoospermic individuals were selected for further analysis. This decision was made because the objective was to identify and investigate variants in OXPHOS genes that could potentially contribute to the reduced sperm motility observed in asthenozoospermic men.

### 2.2. Unique OXPHOS Variants in Asthenozoospermic Men

Out of the 680,099 unique variants found in men with asthenozoospermia, 309 were identified within OXPHOS genes, as shown in Table S1. Table 1 displays the distribution of these variants among the mitochondrial respiratory complexes and their associated genes, with no variants detected in Complex V.

Furthermore, as shown in Table 2, the majority of the unique variants were found in nuclear-encoded OXPHOS genes (94.5%) rather than in mitochondrial-encoded OXPHOS genes (5.5%).

It is worth noting that out of the 309 variants mapped on OXPHOS genes in men with asthenozoospermia, 22 (7.1%) were novel variants.

### 2.3. Unique OXPHOS Variants in Asthenozoospermic Men—Genomic Consequences and Missense Variants

**Figure 1:**
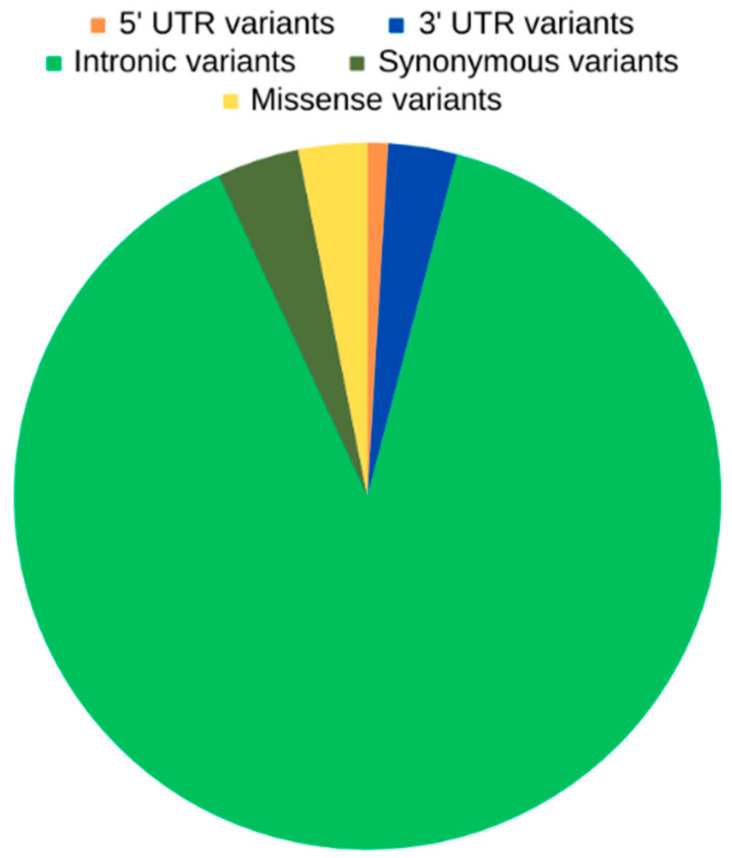
Unique OXPHOS variants in asthenozoospermic men and their genomic consequences.

The unique OXPHOS variants identified in asthenozoospermic men were also categorized based on their genomic consequences. As shown in Figure 1, the majority of variants were intronic (89%), followed by synonymous variants (3.8%), missense variants (3.1%), variants in 3′ untranslated (UTR) regions (3.1%), and variants in 5′ UTR regions (0.9%).

Regarding missense variants, as shown in Table 3, we evaluated them using SIFT [11] and Polyphen2 [12] scores to determine their impact on protein functionality. We found that only one variant, rs35462421, is deemed to have a damaging effect according to both SIFT [11] and Polyphen2 [12]. Table 3 also presents the allele frequencies for the European population, as this study focused on Europeans.

### 2.4. Unique OXPHOS Variants in Asthenozoospermic Men—Variants with Potential Functional Effect

To comprehensively evaluate the functional significance of the unique OXPHOS variants identified in asthenozoospermic men, we utilized two different databases: RegulomeDB [13] and 3DSNP 2.0 [14]. As presented in Table 4, we identified a total of twenty-eight variants that are most likely to have a functional impact, as they have a RegulomeDB rank ranging from 1a to 3b and a 3DSNP score greater than 10.

### 2.5. Unique OXPHOS Variants in Asthenozoospermic Men—Expression Quantitative Trait Loci (eQTL) and Splicing Quantitative Trait Loci (sQTL)

Expression Quantitative Trait Loci (eQTLs) and Splicing Quantitative Trait Loci (sQTLs) are two types of genetic loci that influence gene expression and RNA splicing, respectively, at a quantitative level. Both eQTLs and sQTLs are fundamental in genomics and molecular biology because they offer insights into how genetic variation affects phenotypic variation [15,16,17]. Therefore, we investigated the unique variants identified in OXPHOS genes in asthenozoospermic men using the GTex portal [18]. The identified variants are presented in Table 5, with only the variants associated with eQTLs and sQTLs in testis and prostate tissues being selected.

### 2.6. Unique OXPHOS Variants in Asthenozoospermic Men—Association with Diseases

SNPnexus [19] was utilized to identify the association between unique OXPHOS variants in asthenozoospermic men and diseases, as reported in previous studies. As shown in Table 6, five variants were identified as being associated with Leigh syndrome or/and Mitochondrial complex I deficiency.

### 2.7. Unique OXPHOS Variants in Asthenozoospermic Men—Interactions with miRNAs

MicroRNAs (miRNAs) play a crucial role in gene regulation by targeting messenger RNAs (mRNAs) for degradation or translational repression. They achieve this by binding to complementary sequences within the mRNA [20,21]. Therefore, studying variants that affect miRNA-mRNA interactions is essential because these variations can disrupt the delicate balance of gene expression, ultimately leading to abnormal protein production [22].

To determine if the unique variants in OXPHOS genes have an impact on mRNA-miRNA interactions, miRNASNP v3 [23] was utilized. Table 7 presents the findings, showing that seven variants were identified to affect the binding sites of miRNAs, resulting in either miRNA loss or/and gain. All of these variants were located in the 3’ UTR of OXPHOS genes.

## 3. Discussion

Asthenozoospermia, characterized by reduced sperm motility, stands out as a key contributor to male infertility. The etiology of this condition is closely linked to energy metabolism, as optimal energy production is essential for sperm motility [8]. This underscores the critical role of research into oxidative phosphorylation (OXPHOS) genes, which are fundamental for cellular energy production, in understanding the molecular basis of asthenozoospermia.

More specifically, in the past years, emerging studies have focused on the interplay between OXPHOS, mitochondrial function, and asthenozoospermia. Notably, Barbagallo et al. (2020) [24] observed diminished activities of mitochondrial respiratory complexes I, II, and IV in asthenozoospermic patients compared to fertile men, suggesting a direct link between OXPHOS dysfunction and reduced sperm motility. Furthermore, mutations in mitochondrial DNA (mtDNA) and their association with male infertility, particularly asthenozoospermia, have garnered significant scientific interest [25,26]. Studies also show that key features of non-motile sperm include mitochondrial membrane integrity disruption and compromised sheath function, with mitochondria contributing significantly to movement energy [9,27]. Alterations in mitochondrial chain enzyme activities can impinge on sperm motility too. Research indicates a correlation between mitochondrial enzyme activity, sperm motility, and idiopathic asthenozoospermia suggesting that mitochondrial impairment may be a causative factor [24].

Therefore, pinpointing specific genetic variants within OXPHOS genes linked to decreased sperm motility may unveil new aspects of asthenozoospermia pathophysiology and open avenues for targeted therapeutic interventions.

In the present study, we performed whole genome sequencing on blood samples obtained from asthenozoospermic and normozoospermic men. Our objective was to identify specific genetic variants that were exclusive to either group, as these variants could potentially contribute to the underlying pathology or serve as potential biomarkers. We specifically focused on variants within the OXPHOS pathway, which were found exclusively in asthenozoospermic men. Through the use of a comprehensive set of analytical tools, we identified variants within the OXPHOS genes that are most likely to impact the asthenozoospermic phenotype. More specifically, out of the 680,099 unique variants detected in individuals with asthenozoospermia, 309 were located within OXPHOS genes. Notably, as indicated in Table 8, nine of these variants were deemed high-priority for further investigation due to their significant effects, such as associations with diseases, expression quantitative trait loci (eQTLs), etc., as determined by our in silico analyses.

It should be emphasized that no previous studies have linked the identified prioritized OXPHOS variants with male infertility. According to SNPnexus [19], many of these variants are associated with conditions such as Leigh syndrome or mitochondrial complex I deficiency. Leigh Syndrome is a severe neurological disorder that typically manifests in infancy or early childhood and mitochondrial complex I deficiency is one of the most common biochemical defects observed [28]. Despite these associations, the potential implications of these variants in the context of reproduction, particularly male infertility, remain unexplored.

Regarding the genes on which the prioritized variants were mapped, *NDUFA10* was identified as deregulated in the spermatozoa of first (F1) and second (F2) generation male mice following gestational bisphenol A (BPA) exposure, which was associated with decreased sperm count, motility, and intracellular ATP levels [29]. Similarly, another mouse study demonstrated that benzo[a]pyrene exposure inhibits testosterone through *NDUFA10*-mediated mitochondrial compromise in Leydig cells [30]. Additionally, *NDUFA5* was also found to be downregulated in mouse testes after benzo[a]pyrene exposure [30]. However, BPA exposure significantly increased *NDUFV2* in mouse testes, according to another study [31].

Furthermore, another interesting study in mice revealed a significant difference in the abundance of Ndufv3 between heavy (orthodox) and light (condensed) mitochondria in mouse testis, with condensed mitochondria originating from orthodox ones during meiosis and being essential for acrosomal matrix formation [32]. Lie et al. (2022) [33] reported also that reduced *COX6C* expression led to impaired COX enzyme activity, affecting mitochondrial ATP production and thus sperm motility in buffalo. Moreover, oxidative phosphorylation was found to be enriched in all undifferentiated spermatogonia subtypes studied, with significant differences in the relative abundances of Ndufv1 and Cox6c transcripts between control and E4f1-depleted spermatogonia, the latter condition leading to a progressive loss of undifferentiated spermatogonial cells [34]. Other studies highlighted the differential expression of *COX6C*, *NDUFA5*, *NDUFS7*, and *NDUFV2* between progenitor and differentiating spermatogonia, too [35].

All the above findings indicate that OXPHOS genes play a pivotal role in the regulation of sperm motility, and these insights underline the importance of mitochondrial integrity in sperm development. Therefore, the study of the prioritized reported above could serve as a roadmap for future research.

Furthermore, seven variants were identified to create or disrupt miRNA binding sites, according to miRNASNP v3 [23]. MiRNAs typically bind to complementary sequences within the 3′ untranslated regions (3’ UTRs) of target messenger RNAs (mRNAs), leading to mRNA degradation or inhibition of translation and, thus, downregulation of gene expression. This interaction is highly sequence-specific; therefore, an SNP within the miRNA binding site can significantly alter miRNA binding affinity, resulting in disrupted gene regulation [36]. Although no studies have directly linked the variants reported in Table 7 with gene regulation through miRNAs in asthenozoospermia, several miRNAs presented in this table have been previously associated with male infertility.

Notably, miR-7-1-3p is upregulated in patients with idiopathic azoospermia [37], and a significant negative correlation between its expression levels and sperm concentration has been reported [38]. Similarly, miR-122 has been linked to infertility and identified as a potential sperm quality biomarker [39], with various studies supporting its crucial role in male infertility [38,40,41,42,43]. Furthermore, miR-21 has been shown to regulate the self-renewal of mouse spermatogonial stem cells [44], with potential implications for spermatogenesis also reported in other animal studies [45,46]. MiR-495, recognized also for its role in reproduction through various animal studies [47,48,49], is expressed in the testis [47] and has been associated with the progression of human pregnancy [50], as well as mitochondrial metabolism [51]. Additionally, miR-329 is expressed in rat Leydig cells and plays a role during development from the progenitor to the adult stage [52]. Finally, several miRNAs have been indicated to play a role in female fertility, such as miR-299 [53], the miR-548 family expressed in the female reproductive tract with various regulatory roles [54], miR-589 [55], and miR-1266 and miR-340 associated with pregnancy progression [50].

All the above indicate that miRNAs play a crucial role in male infertility and reproduction in general, and further studies are needed to decipher their specific interactions. It is also paramount for future studies to perform functional experiments to validate the interactions reported here as well as the impact of the reported SNPs in these interactions and to further elucidate the role of the reported miRNAs in conditions such as asthenozoospermia.

In discussing the limitations of our study, it is important to note that the research was conducted on a relatively small cohort. This cohort consisted of five individuals with asthenozoospermia and ten with normozoospermia. We acknowledge that the small sample size may limit the statistical power of our analyses and the generalizability of our findings. This is a critical aspect to consider when interpreting our results, as the conclusions drawn from a limited dataset may not fully represent broader populations. Therefore, we strongly encourage future research to involve larger and more diverse cohorts, which would help replicate and broaden our observations, thereby enhancing the reliability and applicability of our findings. However, it is worth noting that, despite the limited sample size, our study provides valuable preliminary insights into the distinct genomic profile between asthenozoospermia and normozoospermia. This is particularly significant considering the scarcity of studies that specifically examine these specific conditions using next-generation sequencing in this research area. Furthermore, much of the existing research on male infertility also involves relatively small cohorts, similar to ours [56,57,58]. This commonality underscores the broader challenge within the field and emphasizes the urgent need for larger-scale studies. From this perspective, our findings contribute meaningfully to the existing body of literature and serve as a starting point for more extensive future research. Furthermore, we primarily used bioinformatics approaches and in silico analyses to investigate the significance of SNPs within OXPHOS genes in relation to male infertility. Similarly, we employed computational algorithms to predict the interactions between mRNAs and miRNAs (miRNASNP v3 [23]). As these interactions were not validated through experimental procedures, it is possible that some of the miRNA interactions proposed in this study may not be influenced by SNPs in OXPHOS genes, as suggested. However, to mitigate these limitations, we employed whole genome sequencing, which, due to its comprehensive nature, facilitates the examination of the entire genome and provides a thorough overview of the genetic landscape. More importantly, the number of studies that utilize whole genome sequencing to investigate male infertility is limited. Additionally, we leveraged a broad spectrum of databases and varied analytical tools to bolster the robustness of our findings, including SNPnexus [19], RegulomeDB [13], and the GTEx portal [18], among others. We also adhered to stringent selection criteria, particularly with regard to RegulomeDB and 3DSNP scores, to enhance the reliability of our results. Finally, in several instances, we further validated our findings by concurrently utilizing pairs of databases, such as SIFT [11] and Polyphen2 [12], as well as RegulomeDB [13] and 3DSNP 2.0 [14], to ensure the accuracy and consistency of our conclusions.

Thus, regarding future directions, studies involving larger, more diverse cohorts are essential to validate our findings. Genome-wide association studies (GWAS) could also play a crucial role in determining whether the variants identified in this study are associated with asthenozoospermia. Additionally, it is of utmost importance to conduct functional experiments, potentially utilizing knockout models, in order to verify the impact of the reported variants on protein functionality and to comprehend their specific contributions to the asthenozoospermic phenotype. Further investigations are also required to elucidate the involvement of OXPHOS SNPs in male infertility, including an assessment of whether the mutations we have reported exhibit dominant or recessive inheritance patterns. Lastly, it is essential to experimentally validate the predicted interactions, such as those between miRNAs and mRNAs, in order to confirm their biological significance. It would also be interesting to investigate the role of other metabolic pathways, such as glycolysis, in future studies. These pathways are involved in energy production, and thus their disruption can also potentially affect sperm motility. It should also be noted that incorporating Computer-Assisted Sperm Analysis (CASA) and flow cytometry into future genetic studies on male infertility offers a promising avenue that could enhance our understanding of the complex mechanisms underlying this condition. CASA, with its advanced quantitative analysis capabilities, provides a comprehensive evaluation of sperm motility and kinematics. Meanwhile, flow cytometry introduces high-throughput analysis at the cellular level, enabling rapid assessment of sperm cell populations, detection of cellular heterogeneity, and detailed examination of phenotypic characteristics. These advanced techniques hold significant potential for genetic studies, where the complex interplay between genotype and phenotype demands a nuanced understanding of sperm function. They are especially particularly effective when analyzing large datasets, providing a level of detail and efficiency that traditional methods cannot match. However, we should keep in mind that these techniques require specific standardization protocols and extensive training to ensure reliable results.

## 4. Materials and Methods

### 4.1. Patient Recruitment

Human blood and sperm samples were collected from willing volunteers in partnership with the “Embryolab IVF Unit” in Thessaloniki, Greece, as part of the Spermogene research program. Ethical approval was granted by the University of Thessaly Ethics Committee in Volos, Greece, and all participants provided written informed consent. To gather comprehensive information, volunteers completed a questionnaire regarding their health, medical history, and other relevant details.

Exclusion criteria encompassed patients with varicocele, reproductive tract infections, testicular injuries or pathologies, a history of cryptorchidism, orchitis, epididymitis, as well as those with certain systemic diseases, Y microdeletions, or other chromosomal abnormalities. Additionally, all participants self-reported having Greek ethnicity.

All volunteers underwent an andrological examination, and semen analysis was conducted. Sperm samples were collected through masturbation following a minimum abstinence period of two to three days and these were allowed to liquefy at 37 °C for 30 min before analysis. Semen analyses were performed according to the fifth edition of the World Health Organization (WHO) manual for the examination and processing of human semen (available at: https://apps.who.int/iris/handle/10665/44261 (accessed on 5 March 2024)). More specifically, the main analysis (seminogram) was performed using the cell vision counting slides (Tek-Event) for cell counting and observation on Nikon Eclipse TS100, Nikon Eclipse E200, and Nikon Eclipse Ts2 microscopes (Nikon Instruments, Tokyo, Japan). Furthermore, all semen analyses were performed by the same laboratory, undergoing regular internal and external quality controls. The reference values from this edition were used to categorize individuals into normozoospermic and asthenozoospermic phenotypes.

### 4.2. DNA Extraction and Sample Preparation

Genomic DNA was isolated from whole EDTA blood samples using the PureLink Genomic DNA Mini Kit (Invitrogen, Waltham, MA, USA—Catalog number: K182002) following the manufacturer’s instructions. DNA concentration was determined using the Qubit 2.0 fluorometer with the Qubit dsDNA BR Assay Kit (Invitrogen, Waltham, MA, USA—Catalog number: Q32850). The DNA quality was also accessed through agarose gel electrophoresis.

For sample preparation for WGS, three sequencing pools were created. Specifically, DNA obtained from ten normozoospermic individuals was divided into two pools, each comprising DNA from five individuals. Additionally, a third pool was generated using DNA from five asthenozoospermic individuals. The DNAs in each pool were mixed equimolar, resulting in a final concentration of 100 ng/uL and a total quantity of 2 mg.

### 4.3. Whole Genome Sequencing (WGS)

Following the sample preparation, whole genome sequencing was performed by Novogene (Cambridge, UK). Genomic DNA prepared as explained above was utilized to construct libraries for WGS. After normalization and stringent quality control, the qualified libraries were sequenced on an Illumina HiSeq 3000 platform (Illumina Inc., San Diego, CA, USA) as 100-bp paired-end reads. The average sequencing coverage achieved was 30×.

Standard bioinformatics analysis was conducted on the sequencing data. Initially, the quality of the reads was assessed using FASTQC (available online at: http://www.bioinformatics.babraham.ac.uk/projects/fastqc/ (accessed on 5 March 2024)), followed by the removal of low-quality reads (with a minimum PHRED score of 30) and adapter sequences using Trimmomatic (v0.39) [59]. The remaining reads were aligned to the human reference genome (GRCh37/hg19) obtained from the Ensembl database (accessed on 5 March 2024) [60], employing the Burrows-Wheeler aligner (BWA) [61]. Duplicate reads resulting from polymerase chain reaction (PCR) were eliminated using Picard tools, and subsequently, SAM files were converted to BAM files with SAMtools (v1.19.2) [62]. At this point, individual BAM files for normozoospermic pools were merged into a single file. Following this, variant calling was performed using freeBayes (v1.3.7) [63], and the results were stored in variant call format (VCF). BCFtools (v1.17) [62] was utilized to compare VCF files from normozoospermic and asthenozoospermic individuals to identify unique variants specific to each group. Finally, the unique variants were annotated using the VEP tool (available at: https://www.ensembl.org/Tools/VEP (accessed on 5 March 2024)) to gather biological information and predict their effects on protein functionality and pathogenicity.

### 4.4. Investigation of Unique Mutations in OXPHOS Genes—Bioinformatics Approach and Tools

Among the unique variants exclusively found in asthenozoospermic individuals after WGS, those mapped to OXPHOS genes were selected and categorized by type (nuclear or mitochondrial encoded) and OXPHOS complexes (I–V).

Evaluation of these mutations extended to understanding their impact on OXPHOS function and their potential role in asthenozoospermia using various databases. Specifically, RegulomeDB (accessed on 5 March 2024) [13] and 3DSNP 2.0 [14] databases were utilized to assess potential functional effects. RegulomeDB [13] categorizes SNPs based on the presence or absence of functional elements, encompassing protein binding sites, chromatin structure, eQTL (expression quantitative trait loci), histone modifications, and more. Each SNP receives a ranking on a scale from 1 to 7, with lower values signifying a higher likelihood of having a regulatory function. Similarly, the 3DSNP 2.0 [14] is a comprehensive database that consolidates data about 3D-interacting genes, enhancer states, transcription factor binding sites, etc. This information is used to compute a functional score for each SNP, with higher scores signaling a greater probability of SNP functionality. Additionally, information on population genetics was gathered from the 1000 Genomes Project [64], gnomAD [65], and NCBI ALFA (Allele Frequency Aggregator) [66]. SNPnexus [19] provided details about the consequences of unique variants on OXPHOS genes (e.g., intergenic, synonymous, missense, etc.) and their previous associations with other diseases. Additionally, for assessing the impact on protein functionality, Polymorphism Phenotyping v2 (PolyPhen2) [12] and Sorting Intolerant From Tolerant (SIFT) [11] tools were employed. Information on expression quantitative trait loci (eQTL) and splicing quantitative trait loci (sQTL) were obtained through the Genotype-Tissue Expression Project (GTEx) [18]. Finally, miRNASNP v3 [23] was utilized to determine whether the identified variants influenced the binding of noncoding RNAs, potentially affecting the regulation of genes crucial for the OXPHOS machinery.

## 5. Conclusions

In summary, this investigation stands as the first comprehensive study to utilize whole genome sequencing for examining OXPHOS genes and their variants in the context of asthenozoospermia. By identifying 309 OXPHOS gene variants exclusively in asthenozoospermic men, our research offers a pioneering roadmap for future studies on the genetic basis of male infertility. More specifically, the extensive dataset of variants provides invaluable insights into the genetic landscape of asthenozoospermia and the significance of these variants by using different tools and databases. Finally, it not only enhances our understanding of the condition but also sets the foundations for advancements in assisted reproductive technologies (ART) and the development of diagnostic and prognostic tools, as the diverse range of previously unexplored variants identified here presents a rich resource for further study, potentially serving as biomarkers for the diagnosis of male infertility.

## Figures and Tables

**Table 1:** Analysis of unique OXPHOS variants in asthenozoospermic men: Distribution across mitochondrial respiratory complexes and associated genes, gene length to variant ratio, and proportion of OXPHOS variants to total variants.

Genes	Variant Number	Length of Gene/ Variant Number (%)	OXPHOS Variants/Total Variants in Asthenozoospermic (%)
***Mitochondrial Respiratory Complex I***			
*NDUFS1*	3	0.0067	0.0004
*NDUFS2*	3	0.0170	0.0004
*NDUFS3*	1	0.0052	0.0001
*NDUFS7*	7	0.0580	0.0010
*NDUFV1*	1	0.0145	0.0001
*NDUFV2*	5	0.0580	0.0007
*MT-ND2*	1	0.0961	0.0001
*MT-ND5*	10	0.5522	0.0001
*MT-ND6*	1	0.1908	0.0001
*NDUFAB1*	3	0.0196	0.0004
*NDUFA5*	3	0.0143	0.0004
*NDUFA8*	3	0.0191	0.0004
*NDUFA9*	8	0.0177	0.0012
*NDUFA10*	42	0.3151	0.0062
*NDUFA11*	1	0.0079	0.0001
*NDUFA12*	30	0.0277	0.0044
*NDUFA13*	2	0.0150	0.0003
*NDUFB1*	5	0.0863	0.0007
*NDUFB2*	4	0.0125	0.0006
*NDUFB3*	4	0.0279	0.0006
*NDUFB4*	2	0.0324	0.0003
*NDUFB5*	2	0.0087	0.0003
*NDUFB6*	3	0.0149	0.0004
*NDUFB8*	2	0.0319	0.0003
*NDUFB9*	10	0.0241	0.0015
*NDUFB10*	1	0.0410	0.0001
*NDUFC1*	2	0.0056	0.0003
*NDUFC2*	2	0.0173	0.0003
*NDUFS4*	11	0.0090	0.0016
*NDUFS5*	5	0.0601	0.0007
*NDUFS6*	1	0.0068	0.0001
*NDUFV3*	5	0.0149	0.0007
***Mitochondrial Respiratory Complex II***			
*SDHA*	3	0.0077	0.0004
*SDHB*	8	0.0226	0.0012
*SDHC*	11	0.0225	0.0016
*SDHD*	48	0.1446	0.0071
***Mitochondrial Respiratory Complex III***			
*UQCRC2*	4	0.0132	0.0006
*MT-CYB*	2	0.1754	0.0003
***Mitochondrial Respiratory Complex IV***			
*COX5A*	1	0.0055	0.0001
*COX6B1*	8	0.0765	0.0012
*COX6C*	4	0.0195	0.0006
*COX7B2*	34	0.0195	0.0050
*MT-CO2*	2	0.2928	0.0003
*MT-CO3*	1	0.1277	0.0001

**Table 2:** Distribution of unique variants in nuclear- and mitochondrial-encoded OXPHOS genes.

*Mitochondrial Respiratory Complex I*	
Variants in mitochondrial-encoded genes	12
Variants in nuclear-encoded genes	171
***Mitochondrial Respiratory Complex II***	
Variants in mitochondrial-encoded genes	0
Variants in nuclear-encoded genes	70
***Mitochondrial Respiratory Complex III***	
Variants in mitochondrial-encoded genes	2
Variants in nuclear-encoded genes	4
***Mitochondrial Respiratory Complex IV***	
Variants in mitochondrial-encoded genes	3
Variants in nuclear-encoded genes	47

**Table 3:** SIFT [11] and Polyphen2 [12] scores of missense OXPHOS variants identified in asthenozoospermic men.

Genomic Coordinates	Allele	Allele Frequency (Europeans)	Variant	Gene	SIFT Score	Polyphen2 Score
MT:12406-12406	A	0.2%	rs28617389	*MT-ND5*	0.45 (tolerated)	0 (benign)
MT:13708-13708	A	11.6%	rs28359178	*MT-ND5*	0.26 (tolerated)	0 (benign)
MT:13780-13780	G	2.9%	rs41358152	*MT-ND5*	0.01 (deleterious)	0.003 (benign)
MT:13928-13928	C	0.2%	rs28359184	*MT-ND5*	1 (tolerated)	0.18 (benign)
MT:14178-14178	C	0.2%	rs28357671	*MT-ND6*	0.4 (tolerated)	0.023 (benign)
MT:14793-14793	G	3.7%	rs2853504	*MT-CYB*	0.04 (deleterious)	0.003 (benign)
MT:9477-9477	A	8.5%	rs2853825	*MT-CO3*	0.1 (tolerated)	0 (benign)
2:240951071-240951071	T	1.1%	rs35462421	*NDUFA10*	0.01 (deleterious)	0.995 (probably damaging)
16:21976762-21976762	A	4.3%	rs4850	*UQCRC2*	0.04 (deleterious)	0.003 (benign)
5:52942083-52942083	C	96%	rs31304	*NDUFS4*	-	0 (unknown)

**Table 4:** Unique OXPHOS variants in asthenozoospermic men with potential functional significance according to RegulomeDB [13] and 3DSNP 2.0 [14] databases.

Genomic Coordinates	Allele	Allele Frequency (Europeans)	Variant	Gene	Genomic Consequences	RegulomeDB Rank	3DSNP Score
19:1394865-1394865	C	2.1%	rs73515054	*NDUFS7*	3′ UTR variant, intron variant	2b	13.72
9:124897110-124897110	T	8.7%	rs11998959	*NDUFA8*	Intron variant	1f	36.76
9:124897088-124897088	T	8.3%	rs11998958	*NDUFA8*	Intron variant	1f	36.36
7:123197559-123197559	C	8.6%	rs17146099	*NDUFA5*	5′ UTR variant, intron variant	1f	146.4
2:240897460-240897460	C	3.5%	rs7588974	*NDUFA10*	3′ UTR variant, intron variant	2b	10.56
16:2011653-2011667	CCCCCA	0.03%	rs774819361	*NDUFB10*	Intron variant	2a	103.27
8:125551858-125551858	G	3.5%	rs72713101	*NDUFB9*	Intron variant	1f	108.59
8:125554452-125554452	T	3.3%	rs111795428	*NDUFB9*	Intron variant	1f	11.6
8:125552526-125552527	-	3.3%	rs112295879	*NDUFB9*	Intron variant	1b	116.14
11:77790158-77790158	AAAAA	0.1%	rs752264424	*NDUFC2*	Intron variant	2b	104.37
1:161175652-161175652	A	1.8%	rs145629160	*NDUFS2*	Intron variant	1f	13.25
1:161171736-161171736	G	1.8%	rs115518404	*NDUFS2*	Intron variant	1b	146.84
21:44313221-44313221	C	20.2%	rs35197797	*NDUFV3*	Intron variant	1a	211.4
8:100903890-100903890	G	14.1%	rs12544943	*COX6C*	Intron variant	1f	66.33
11:67374581-67374581	C	38.2%	rs1871043	*NDUFV1*	Intron variant	1f	208.1
18:9119489-9119489	T	9.1%	rs41274300	*NDUFV2*	Synonymous variant	1f	28.68
14:92586558-92586558	A	16.3%	rs79507139	*NDUFB1*	Intron variant	1f	16.69
12:95376507-95376507	T	9.2%	rs4923659	*NDUFA12*	Intron variant	1b	16.37
12:95371804-95371806	-	9.2%	rs113060515	*NDUFA12*	Intron variant	1f	13.75
12:95374449-95374449	C	9.2%	rs76835653	*NDUFA12*	Intron variant	1b	59.71
12:95397275-95397275	T	10.1%	rs17321986	*NDUFA12*	Intron variant	1b	201
11:112044398-112044398	C	22.9%	rs12420476	*SDHD*	Intron variant	1f	11.55
11:112034062-112034063	AA	22%	rs5744230	*SDHD*	Intron variant	1d	33.1
11:112037730-112037730	A	10.8%	rs72992972	*SDHD*	Intron variant	1d	14.26
11:112047061-112047061	A	12.2%	rs10431036	*SDHD*	Intron variant	1f	20.65
11:112043614-112043614	A	12.2%	rs11214108	*SDHD*	Intron variant	1f	12.25
11:112048051-112048051	Τ	22.7%	rs7121554	*SDHD*	Intron variant	1f	12.03
11:111991866-111991868	-	0.3%	rs1453244355	*SDHD*	Intron variant	2b	11.47

**Table 5:** Unique OXPHOS variants in asthenozoospermic identified as eQTL and sQTL according to the GTex portal [18].

Genomic Coordinates	Allele	Allele Frequency (Europeans)	Variant	Gene	Genomic Consequences	Function	*p*-Value
7:123197559-123197559	C	8.6%	rs17146099	*NDUFA5*	5′ UTR variant, intron variant	eQTL (Testis)	0.000089
7:123197559-123197559	C	8.6%	rs17146099	*NDUFA5*	5′ UTR variant, intron variant	sQTL (Testis)	9.3 × 10^−8^
7:123190928-123190928	T	8.6%	rs34225533	*NDUFA5*	Intron variant	eQTL (Testis)	0.000036
7:123190928-123190928	T	8.6%	rs34225533	*NDUFA5*	Intron variant	sQTL (Testis)	9.8 × 10^−7^
2:240872465-240872465	A	14.7%	rs11684384	*NDUFA10*	Intron variant	eQTL (Testis)	8.4 × 10^−10^
2:240872465-240872465	A	14.7%	rs11684384	*NDUFA10*	Intron variant	eQTL (Prostate)	2.1 × 10^−15^
21:44325525-44325525	T	20.2%	rs8134542	*NDUFV3*	Intron variant	eQTL (Prostate)	3.9 × 10^−12^
21:44328278-44328278	A	20.2%	rs35893787	*NDUFV3*	Intron variant	eQTL (Prostate)	7.8 × 10^−13^
21:44313221-44313221	C	20.2%	rs35197797	*NDUFV3*	Intron variant	eQTL (Prostate)	9.5 × 10^−13^
8:100894978-100894986	AAAC	18.2%	rs71274941	*COX6C*	Intron variant	sQTL (Testis)	1.1 × 10^−59^
8:100894978-100894986	AAAC	18.2%	rs71274941	*COX6C*	Intron variant	sQTL (Prostate)	1.4 × 10^−28^
8:100903890-100903890	G	14.1%	rs12544943	*COX6C*	Intron variant	sQTL (Testis)	9.1 × 10^−36^
8:100903890-100903890	G	14.1%	rs12544943	*COX6C*	Intron variant	sQTL (Prostate)	1.8 × 10^−16^
11:67374581-67374581	C	38.2%	rs1871043	*NDUFV1*	Intron variant	eQTL (Prostate)	7.6 × 10^−9^
4:46775623-46775623	G	4.7%	rs78130313	*COX7B2*	Intron variant	eQTL (Testis)	0.000032
4:46908004-46908004	A	5.4%	rs371114117	*COX7B2*	Intron variant	eQTL (Testis)	0.00010
4:46908004-46908004	A	5.4%	rs371114117	*COX7B2*	Intron variant	sQTL (Testis)	3.9 × 10^−7^
12:95387542-95387542	Τ	44.4%	rs4923660	*NDUFA12*	Intron variant	eQTL (Testis)	0.000015
12:95387542-95387542	Τ	44.4%	rs4923660	*NDUFA12*	Intron variant	sQTL (Testis)	0.0000037

**Table 6:** Association of unique OXPHOS variants in asthenozoospermic with diseases according to SNPnexus [19].

Genomic Coordinates	Allele	Allele Frequency (Europeans)	Variant	Gene	Genomic Consequence	Association with Diseases
19:1391059-1391059	T	1.9%	rs2074896	*NDUFS7*	intron variant	Leigh syndrome, Mitochondrial complex I deficiency (Benign/Likely benign)
2:240897460-240897460	C	3.5%	rs7588974	*NDUFA10*	3′ UTR variant, intron variant	Leigh syndrome, Mitochondrial complex I deficiency
2:240951071-240951071	T	1.1%	rs35462421	*NDUFA10*	Missense variant	Leigh syndrome (Benign/Likely benign)
5:52942083-52942083	C	96%	rs31304	*NDUFS4*	Synonymous variant	Leigh syndrome, Mitochondrial complex I deficiency (Benign)
18:9119489-9119489	T	9.1%	rs41274300	*NDUFV2*	Synonymous variant	Mitochondrial complex I deficiency (Benign/Likely benign)

**Table 7:** Unique OXPHOS variants in asthenozoospermic men leading to miRNA binding site gain and/or loss according to miRNASNP v3 [23].

Genomic Coordinates	Allele	Allele Frequency (Europeans)	Variant	Gene	miRNA Loss	miRNA Gain
3:120320652-120320652	C	0.7%	rs190013694	*NDUFB4*	hsa-miR-1273h-3p, hsa-miR-1245b-3p, hsa-miR-5700, hsa-miR-3678-3p	hsa-miR-1193, hsa-miR-105-3p, hsa-miR-4754, hsa-miR-6850-5p
19:1394865-1394865	C	2.1%	rs73515054	*NDUFS7*	hsa-miR-495-3p, hsa-miR-5688, hsa-miR-7-2-3p, hsa-miR-589-3p, hsa-miR-7-1-3p, hsa-miR-4773	hsa-miR-2278, hsa-miR-548p, hsa-miR-6501-3p
7:123180937-123180942	GCG	0.6%	rs201784621	*NDUFA5*	hsa-miR-4536-3p, hsa-miR-4787-3p	hsa-miR-8064, hsa-miR-6821-5p, hsa-miR-4783-5p
2:240897460-240897460	C	3.5%	rs7588974	*NDUFA10*	hsa-miR-3155b, hsa-miR-3155a, hsa-miR-4518, hsa-miR-1266-5p, hsa-miR-484, hsa-miR-3664-3p	hsa-miR-6829-3p, hsa-miR-6741-3p, hsa-miR-6778-3p, hsa-miR-6791-3p
12:4798415-4798415	Τ	0.1%	rs181096156	*NDUFA9*	hsa-miR-4712-3p, hsa-miR-580-3p, hsa-miR-539-5p	hsa-miR-577
4:46736853-46736853	Τ	13.4%	rs11736008	*COX7B2*	-	hsa-miR-12135, hsa-miR-4748, hsa-miR-299-5p, hsa-miR-548m, hsa-miR-4464, hsa-miR-548at-5p, hsa-miR-561-3p, hsa-miR-329-5p
11:111966122-111966122	G	0.7%	rs184654032	*SDHD*	hsa-miR-3120-5p, hsa-miR-200a-3p, hsa-miR-1208, hsa-miR-6757-3p, hsa-miR-141-3p, hsa-miR-6760-3p	hsa-miR-340-3p, hsa-miR-122b-3p, hsa-miR-6827-3p, hsa-miR-21-3p

**Table 8:** Prioritized OXPHOS variants for further investigation.

Variant	Gene	Allele Frequency (Europeans)	Missense Variant	Functional Significance	Association with Diseases	eQTLs/sQTLs	miRNA Interactions
rs35462421	*NDUFA10*	1.1%	Damaging according to both databases	-	✓	-	-
rs31304	*NDUFS4*	96%	Unknown impact	-	✓	-	-
rs73515054	*NDUFS7*	2.1%	-	✓	-	-	✓
rs17146099	*NDUFA5*	8.6%	-	✓	-	✓	-
rs7588974	*NDUFA10*	3.5%	-	✓	✓	-	✓
rs35197797	*NDUFV3*	20.2%	-	✓	-	✓	-
rs12544943	*COX6C*	14.1%	-	✓	-	✓	-
rs1871043	*NDUFV1*	38.2%	-	✓	-	✓	-
rs41274300	*NDUFV2*	9.1%	-	✓	✓	-	-

## Data Availability

Whole-genome sequencing data of normozoospermic men used in this study are available through SRA (BioProject ID PRJNA875412, http://www.ncbi.nlm.nih.gov/bioproject/875412 (accessed on 5 March 2024)).

## Supplementary Materials

The following supporting information can be downloaded at: https://www.mdpi.com/article/10.3390/ijms25074121/s1.
